# Sodium Alginate Microneedles Loaded with Vancomycin for Skin Infections

**DOI:** 10.3390/jfb15110316

**Published:** 2024-10-25

**Authors:** Juhaina M. Abu Ershaid, Han Zhang, May Tayyem, Akmal H. Sabri, Ryan F. Donnelly, Lalitkumar K. Vora

**Affiliations:** 1 School of Pharmacy, Queen’s University Belfast, 97 Lisburn Road, Belfast BT9 7BL, UK; juhaina.abuershaid@iu.edu.jo (J.M.A.E.); hzhang24@qub.ac.uk (H.Z.); akmal.sabri@nottingham.ac.uk (A.H.S.); 2 School of Pharmacy, Department of Applied Pharmaceutical Sciences and Clinical Pharmacy, Isra University, Amman 11622, Jordan; 3 School of Pharmacy, Department of Pharmaceutical Technology and Cosmetics, Middle East University, Airport Road, Amman 11831, Jordan; mtayyem@meu.edu.jo; 4 School of Pharmacy, University of Nottingham, University Park, Nottingham NG7 2RD, UK

**Keywords:** microneedles, vancomycin, sodium alginate, MRSA, skin infections

## Abstract

Background: Skin and soft tissue infections (SSTIs) present significant treatment challenges. These infections often require systemic antibiotics such as vancomycin, which poses a risk for increased bacterial resistance. Topical treatments are hindered by the barrier function of the skin, and microneedles (MNs) offer a promising solution, increasing patient compliance and negating the need for traditional needles. Methods: This study focused on the use of sodium alginate MNs for vancomycin delivery directly to the site of infection via a cost-effective micromolding technique. Dissolving polymeric MNs made of sodium alginate and loaded with vancomycin were fabricated and evaluated in terms of their physical properties, delivery ability, and antimicrobial activity. Results: The MNs achieved a 378 μm depth of insertion into ex vivo skin and a 5.0 ± 0 mm zone of inhibition in agar disc diffusion assays. Furthermore, in ex vivo Franz cell experiments, the MNs delivered 34.46 ± 11.31 μg of vancomycin with around 35% efficiency, with 9.88 ± 0.57 μg deposited in the skin after 24 h. Conclusions: These findings suggest that sodium alginate MNs are a viable platform for antimicrobial agent delivery in SSTIs. Future in vivo studies are essential to confirm the safety and effectiveness of this innovative method for clinical use.

## 1. Introduction

Methicillin-resistant *Staphylococcus aureus* (MRSA) is a strain of the common bacterium *Staphylococcus aureus* that is a gram-positive bacterium [1,2]. This strain of bacteria typically results in infections of varying severity upon ingress into the skin, underlying tissues, or bloodstream. This could occur in the case of wounds, chronic skin conditions, or surgical intervention [3,4]. Shortly after the discovery of the narrow-spectrum β-lactam antibiotic methicillin, MRSA infection emerged in England in 1961 as a result of poor antimicrobial stewardship [5]. MRSA is considered one of the most common pathogenic strains of *Staphylococcus aureus* and has severe clinical efficacy in patients with compromised immune systems [6,7]. However, the reach of MRSA extended beyond the limits of hospitals, as it infected otherwise healthy individuals with no previous hospital admission. This has raised alarm bells among medical professionals, urging them to intensify efforts in understanding and combating this pathogen [8].

MRSA has evolved remarkably owing to the selective evolutionary pressure imposed by the impetuous use of antibiotics, culminating in an increase in the number of bacterial strains that are capable of resisting several classes of antibiotics [9]. This resistance is challenging and a threat to public health at the global scale. In addition, MRSA ingress into the skin as a result of trauma or injury can lead to skin and soft tissue infections (SSTIs), which can range from mild and manageable to life-threatening and debilitating [10].

Several types of antibiotics, which are glycopeptide antibiotics, have been indicated for the treatment of SSTIs caused by MRSA caused by vancomycin; these antibiotics are used as a first-line choice for severe or complicated SSTIs when MRSA is suspected or confirmed [11]. Vancomycin exhibits high potency against MRSA by inhibiting bacterial cell wall synthesis [12]. This is achieved as the antibiotic molecule binds to the D-Ala-D-Ala terminus of the peptidoglycan precursors, preventing their incorporation into the cell wall. This unique mechanism makes vancomycin effective against MRSA strains that have acquired resistance to other beta-lactam antibiotics [13].

With the rise of MRSA and concerns about vancomycin resistance, researchers continue to explore new treatment avenues for SSTIs. This includes the development of novel antibiotics and combination therapies. Vancomycin is a hydrophilic glycopeptide with a molecular weight of 1449 kDa and a Log P of −3.1 [14]. The inherent physiochemical properties of vancomycin prevent passive permeation of the glycopeptide through the skin layers, thus preventing the antibiotic from reaching the desired target site. Owing to this limitation, alternative formulation strategies that could augment the permeation of vancomycin into the skin for the treatment of MRSA-based SSTIs with minimal toxicity are needed [15]. One approach involves the use of MNs. MNs are micron-scale projections on a flat baseplate that are capable of breaching the outermost layer of the skin, the *stratum corneum*, in a minimally invasive and transient fashion [16]. This allows the antibiotic to reach the site of infection effectively and minimises systemic exposure [17]. This targeted delivery of vancomycin eliminates the risk of several toxicities associated with intravenous vancomycin, including nephrotoxicity and ototoxicity [18].

Vancomycin-loaded MNs should be formulated considering several aspects, including maintaining drug activity after release from the MNs and using biocompatible materials to avoid adverse reactions or tissue damage [19]. Sodium alginate is a natural biopolymer extracted from brown algae. It has been widely applied in various applications, including the pharmaceutical and biomedical fields, owing to its biocompatibility, nontoxicity, and ease of gelation [20]. Additionally, it has been used to formulate MNs due to several advantages, including its ability to encapsulate a wide range of molecules by forming a stable hydrogel [21]. These hydrogels entrap drugs within the MN structure while releasing them upon contact with body fluids from the hydrogel network in a sustained fashion [22]. Sodium alginate is a biocompatible and biodegradable polymer that is commonly combined with other polymers to enhance the physical properties of MNs. The addition of sodium alginate in the preparation of MNs endows the formulation with increased mechanical strength, increased drug loading capacity, and the ability to tailor the rate of drug release [23]. In this study, sodium alginate was used to formulate dissolving MNs loaded with vancomycin. A light microscope was used to visualise and evaluate the physical appearance of the MNs. Furthermore, a TA-X2 Texture Analyser was used to evaluate the mechanical and insertion ability of the MNs. Finally, the antimicrobial activity of the vancomycin MNs was inspected via a disk diffusion test. This work aimed to determine the feasibility of applying MNs for the treatment of skin infections.

## 2. Materials

Vancomycin hydrochloride was purchased from Alfa Aesar (Lancashire, UK). Alginic acid sodium salt powder, citric acid, gelatin, Muller Hinton broth (MHB), Muller Hinton agar (MHA), polyethylene glycol (PEG) (M.W. 400 D), polyvinyl alcohol (PVA) (M.W. 9–10 kDa), polyvinyl alcohol (PVA) (M.W. 85–124 kDa), sodium chloride, acetonitrile (ACN) (>99.9%), and methanol (MeOH) (>99.9%) were purchased from Sigma-Aldrich (Dorset, UK). Glycerol (99.5%) was purchased from VWR International (Leicestershire, UK). Phosphate-buffered saline (PBS) tablets (pH 7.4) were purchased from Oxoid Limited (Hampshire, UK). Phosphoric acid (85%) and triethylamine anhydrous were purchased from Fluorochem (Hadfield, Old Glossop, UK). *Staphylococcus aureus* 29213, *Streptococcus epidermidis* 11047, and MRSA 33592 were purchased from ATCC. HPLC-grade water was obtained from a water purification system (Elga PURELAB^®^ DV 25, Veolia Water Systems, Kilkenny, Ireland). All the other chemicals were of analytical reagent grade.

## 3. Methods

### 3.1. MNs Preparation Method

**Figure 1:**
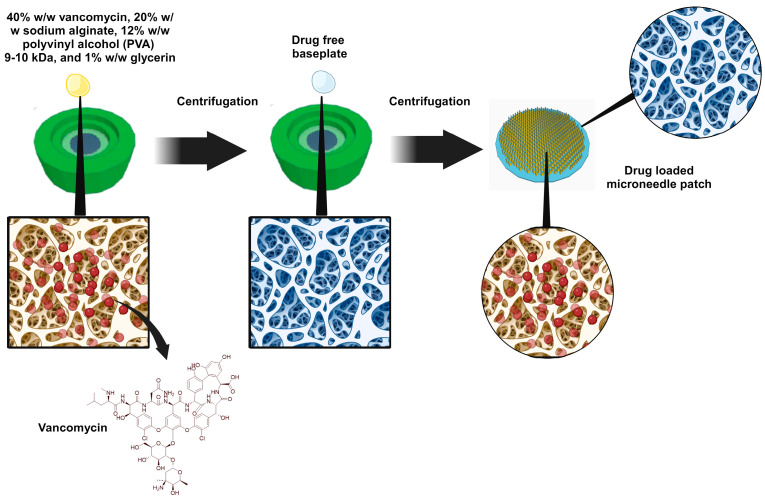
Preparation method of sodium alginate microneedles loaded with vancomycin.

Circular MNs moulds (600 pyramidal MNs per array with areas of 0.75 cm^2^ and 750 μm MN heights) were used to prepare MNs via the casting method. As shown in Figure 1, 300 μL of an aqueous solution containing 40% *w*/*w* vancomycin, 20% *w*/*w* sodium alginate, 12% *w*/*w* polyvinyl alcohol (PVA) 9–10 kDa, and 1% *w*/*w* glycerin was cast into the MN moulds. The MNs were subsequently centrifuged at 6000× *g* for 5 min and left to dry at room temperature (RT) for 24 h after the excess formulation was removed. A base plate was formulated by adding a drug-free polymeric solution of 20% *w*/*w* sodium alginate, 12% *w*/*w* polyvinyl alcohol (PVA) 9–10 kDa, and 1% *w*/*w* glycerin into the MN moulds. The MNs were subsequently centrifuged at 6000× *g* for 5 min and left to dry at room temperature for 24 h.

### 3.2. MN Characterisation

MNs were characterised in terms of their morphology, needle length, strength, insertion ability, and drug content. A light microscope was used to visualize the microneedles and to check their full formation. MN lengths were also recorded, and the average length was calculated.

A TA-X2 Texture Analyser (Stable Microsystem, Haslmere, UK) was used to perform compression force tests and penetration tests. These tests are commonly conducted to evaluate the mechanical strength and penetration ability of MNs [24]. The movable cylindrical probe of the texture analyser was used to move the MNs at a speed of 0.5 mm per second downward into a metallic surface, and a force of 32 N was applied for 30 s [25]. The height reduction percentage was calculated according to Equation (1).

(1) $$Height\;reduction\%=\frac{Hb-Ha}{Hb}\times 100\%$$

Hb: The original average length of the MNs before compression.

Ha: Average length of MNs after compression.

To evaluate the insertion ability of the MNs, Parafilm^®^M was folded into eight layers to obtain an average thickness of approximately 1 mm. The same movable cylinder with the same settings was used to move the MNs toward the eight layers of Parafilm^®^M. A stereomicroscope was used to count the holes in each layer. The percentage of insertion was calculated according to Equation (2).

(2) $$Holes\;created\%=\frac{Number\;of\;holes\;in\;each\;Parafilm®.M\;layer}{Total\;number\;of\;MNs}\times 100\%$$

To quantify vancomycin in this work, a previously described pharmaceutical analytical method was validated via ultraviolet-HPLC [26]. A Phenomenex^®^ C_18_ column (4.6 cm × 150 mm, 5.0 µm) was used as the stationary phase, while the mobile phase was composed of phosphate buffer and a mixture of methanol and acetonitrile. Detection was carried out at a wavelength of 240 nm at 20 °C with a flow rate of 0.4 mL/min and an injection volume of 50 μL. The vancomycin content was determined by dissolving the MNs in 20 mL of PBS at 37 °C. The solution was then filtered through a 0.22 µm filter membrane and diluted with PBS. Finally, the samples were analysed using a validated ultraviolet-high-performance liquid chromatography (UV-HPLC) method.

### 3.3. Attenuated Total Reflectance-Fourier Transform Infrared Spectroscopy

An attached total reflection-Fourier transform infrared (ATR-FTIR) instrument with MIRacle™ software (Pike Technologies Ltd., Madison, WI, USA). was used to evaluate the possible interactions between vancomycin, sodium alginate, and the other polymers used. The samples (vancomycin, physical mixture, and MNs) were placed independently under a digital torque controller on the sample stage holder, with wavenumbers ranging between 600 cm^−1^ and 4000 cm^−1^ with a resolution of 4.0 cm^−1^. MIRacle™ software was used to obtain the infrared spectrum.

### 3.4. Ex Vivo Skin Deposition Study

An ex vivo skin deposition study using a Franz diffusion cell apparatus was performed to evaluate the ability of MNs to deposit vancomycin into full-thickness neonatal porcine skin [27]. Vancomycin quantities in the reservoir compartment of the Franz diffusion cells and in the neonatal porcine skin were determined via validated UV-HPLC methods. Piglets were provided by the Agri-Food and Bioscience Institute (Hillsborough, Northern Ireland, UK). The reservoir compartment was filled with PBS solution (pH = 7.4). Before the experiment was performed, the skin was equilibrated in PBS (pH = 7.4), the skin hair was shaved, and the skin was rinsed again with PBS (pH = 7.4). The skin pieces were dried on paper towels. The skin was attached to the donor compartment of Franz cells with cyanoacrylate^®^ glue. The MN arrays were inserted into the centre of the skin and gently pressed with fingers for 30 s by pushing the flat end of the syringe plunger onto the base plate of the MNs. A cylindrical metal with a diameter of 11.0 mm and a weight of 5.0 g was placed on the top of the MNs for fixation during the experiment. One layer of Parafilm^®^M was placed on top of the donor chamber and on the receptor arm to prevent fluid evaporation from the 12 mL receptor compartment. The temperature was maintained at 37 ± 1 °C. At predetermined time intervals (3, 5 and 24 h), a 200 μL sample was taken from the sampling arm, and the sample volume was replaced with fresh release media to maintain the sink condition. The samples were mixed with 0.5 mL of methanol and centrifuged for 30 min for analysis via the validated HPLC method. After 24 h, the skin was removed from the Franz cell compartment, the MNs on the surface were scraped off, and the skin was placed in an Eppendorf^®^ tube containing 1 mL of water with a metal bead. The drug was extracted at 50 Hz by using Tissue Lysser^®^ for 15 min and centrifuged at 6000× *g* for 10 min. The supernatants were collected and filtered through a 0.2 μL filter. All the samples were appropriately diluted and analysed using HPLC.

### 3.5. Antimicrobial Activity

#### 3.5.1. Inoculum Preparation

The bacterial cells were maintained on cryoconervative beads at −80 °C in 10% glycerol. The day before the experiment, a few beads were inoculated in MHB and cultured in a shaker incubator at 37 °C at 100 rpm overnight. The supernatant was discarded after the bacterial culture was centrifuged at 3000 rpm for 12 min. The pellets were subsequently suspended in PBS, such that the optical density at 550 nm (OD_550_) was 0.3 for *S. aureus, S. epidermidis*, and MRSA; the approximate inoculum density was 1 × 10^8^ CFU/mL, as verified by the viable count. Then, 5 mL was added to 99.5 mL of MHB and incubated in a shaker incubator at 37 °C at 100 rpm for 3 h until the bacteria reached the exponential phase of growth.

#### 3.5.2. Determination of Antimicrobial Activity of Vancomycin

The minimum inhibitory concentration (MIC) and minimum biocidal concentration (MBC) were determined according to the CLSI broth microdilution method [28].

Stock solutions of vancomycin were prepared in MHB at a concentration of 100 µg/mL. Bacterial cultures were prepared as previously described. After reaching the logarithmic phase of growth, the cultures were cultured in PBS to an OD_550_ of 0.3, and then diluted again in MHB to obtain a concentration of 1 × 10^6^ CFU/mL, as verified by a viable count. MHB (50 µL) was added to the wells of round-bottomed 96-well plates (rows 2 to 10) in triplicate. The vancomycin stock solution (100 µL) was added to the first row of the plate. After that, twofold serial dilutions were performed through the tenth row, and 50 µL was discarded from the final wells. Then, 50 μL of diluted bacterial culture was added to each well for a final inoculum density of 5 × 10^5^ CFU/mL. A positive control mixture containing 50 µL of MHB and 50 µL of bacterial inoculum was included in this experiment, and a negative control mixture containing 100 µL of free MHB was also included. The plates were incubated overnight at 37 °C at 100 rpm in a shaker incubator, after which the MIC was determined as the lowest concentration at which negative growth was observed visually. Each test was repeated three separate times. MBC values were determined by spreading 10 µL from each well. The agar plates were incubated for 24 h in a static incubator at 37 °C, after which 99.9% of the bacteria were killed. The lowest concentration showing no growth was considered the MBC.

#### 3.5.3. Evaluating the Antimicrobial Activities of the Vancomycin MNs via the Disk Diffusion Method

The zones of inhibition were determined according to the CLSI broth microdilution method [29,30]. Bacterial cultures were prepared as previously described and diluted in PBS to 1 × 10^8^ CFU/mL. The cultures were streaked and placed on sterile cotton swabs on sterile MHA plates. The MN arrays were placed on the middle of the MHA plates and gently pressed via sterile tweezers. Plates with negative controls were also included, and unloaded MNs were used to exclude any antimicrobial activity for free MNs. The plates were incubated inverted in a static incubator for 24 h at 37 °C. The next day, the zones of inhibition were measured via a ruler, and the diameters were recorded in cm. Each experiment was carried out on three separate occasions.

### 3.6. Statistical Analysis

The statistical analysis in this study was performed via SPSS 23 software. The data were processed via Microsoft^®^ Excel^®^ 2016. Independent sample t tests were used for comparing groups, and the significance level was set at *p* < 0.05.

## 4. Results and Discussion

### 4.1. MN Characterisation

**Figure 2:**
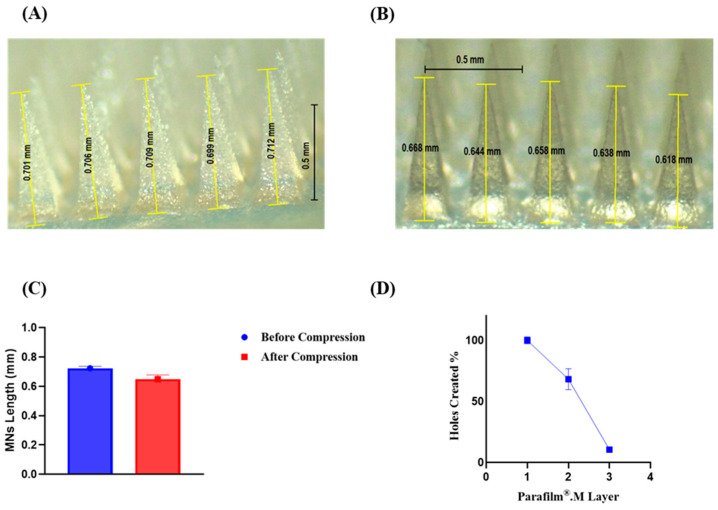
(**A**): Light microscope image of the prepared microneedles before performing the compression force test. (**B**): Light microscope image showing the length of the microneedles after performing the compression force test. (**C**): Microneedle length before and after reduction. (**D**): % Holes created in each layer of Parafilm^®^M upon conducting the Parafilm^®^M insertion test.

In the present work, sodium-alginate-based MNs loaded with vancomycin were fabricated via two-step micromolding techniques, which are commonly employed in MN fabrication [31]. As illustrated in Figure 2, visual inspection via light microscopy revealed that the MNs were fully formed with no bubbles, precipitation, or separation. The MNs were uniform and clear, with an average length of 724 ± 130 μm. These findings indicate the reproducibility of the proposed preparation method. When subjected to an axial force of 32 N, analogous to thumb pressure applied for 30 sec on MNs, the fabricated MNs exhibited a height reduction of 10.23 ± 4.82%. This height reduction percentage is comparable to that reported for other MNs. This finding indicated that the fabricated MNs would not fracture or buckle upon application into the skin [32,33]. The penetration profile of the MNs was subsequently evaluated via a Parafilm^®^M insertion test [24]. A light microscope was used to determine the percentage of holes created in each of the Parafilm^®^M groups, as shown in Figure 2. All the MNs were able to penetrate the first layer of Parafilm^®^M, resulting in 100% of the holes being created in the first layer. Approximately 68% of the holes were found in the second layer, whereas 10% of the holes were created in the third layer. The MNs were able to pierce the third layer of Parafilm^®^M. Each layer of Parafilm^®^M has a thickness of approximately 126 μm, and three layers penetrate to an estimated insertion depth of 378 μm. There were no observable holes within the remaining layers. These findings are in parallel with several studies reporting MNs with similar insertion abilities and successful insertion in vivo. This insertion profile indicates that MNs are capable of piercing the outermost layer of the skin, the *stratum corneum,* with high reproducibility and efficiency, enabling the implantation of drug-laden shafts into the aqueous dermis where they are deposited and released [34]. In addition, vancomycin concentrations were quantified via a previously described UV-HPLC method [35]. Vancomycin was loaded only in the tips of the MNs with a drug-free baseplate, resulting in an overall drug loading of 100 ± 0.165 μg per MN array.

### 4.2. Attenuated Total Reflectance—Fourier Transform Infrared Spectroscopy

**Figure 3:**
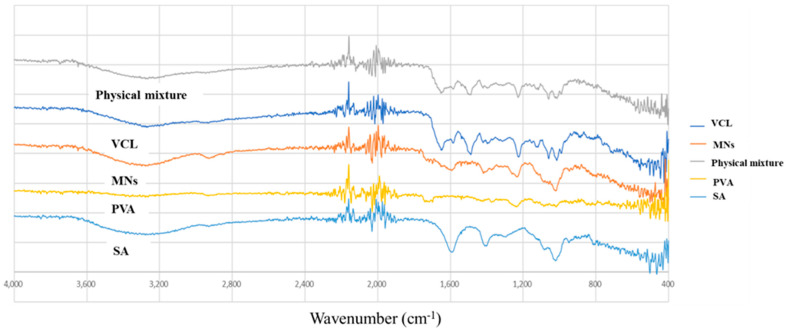
Fourier transmission infrared spectra of sodium alginate (SA), polyvinyl alcohol (PVA), vancomycin (VCL), vancomycin-loaded MNs (MNs), and the physical mixture of these constituents.

An ATR-FTIR study was carried out to investigate the interaction between vancomycin and the excipients used to fabricate the MNs. Figure 3 displays the FTIR spectra of the MNs, vancomycin, sodium alginate, PVA 9–10 kDa, and physical mixture of the polymers. On the basis of these spectra, the characteristic functional groups of each compound are summarised in Table 1. For sodium alginate, the peak at 1591 cm^−1^ is attributed to the asymmetric stretching of carboxylate O-C-O vibrations, the peak at 1401 cm^−1^ may be the deformation vibration of C-OH or the symmetric stretching of carboxylate O-C-O, and the peak at 1 may be the stretching vibration of C-O [36,37]. For PVA, the peak at 1699 cm^−1^ is the stretching vibration of C=O, the asymmetric stretching vibration of C-O-C at 1246 cm^−1^, and the peak at 1035 cm^−1^ is the stretching vibration of C-O or the symmetric stretching vibration of C-O-C [38,39]. For vancomycin, 1651 cm^−1^ is the stretching vibration of C=O, 1488 cm^−1^ is the skeleton vibration of benzene C=C, and the peaks of phenol at 1225 cm^−1^ and 1035 cm^−1^ are the stretching vibrations of C-O [40,41]. The MNs and the physical mixture have characteristic peaks of all the components, as demonstrated below. These findings indicate that MN fabrication did not alter the structure of vancomycin, as the characteristic peaks of vancomycin remained unchanged.

### 4.3. Ex Vivo Skin Deposition Study

**Figure 4:**
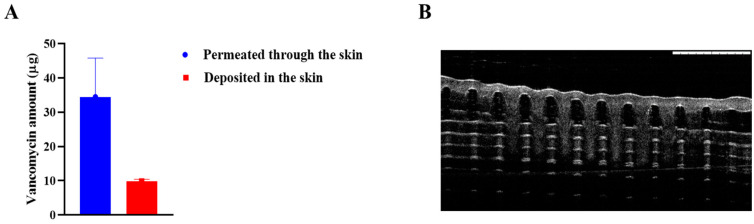
(**A**): Ex vivo skin deposition study of vancomycin-loaded sodium alginate microneedles performed via Franz diffusion cells on full-thickness neonatal porcine skin. (**B**): Optical coherence tomography (OCT) real-time image showing the full insertion of vancomycin-loaded sodium alginate microneedles into the Parafilm^®^ M layers.

Following the physical and mechanical evaluation of the MNs, the ability of the MNs to penetrate, dissolve, and release vancomycin into the skin was evaluated via a Franz cell setup. As illustrated in Figure 4, OCT images were taken while the MNs were inserted into the Parafilm^®^M layers to determine the ability of the MNs to insert. Upon application to the skin, the amount of vancomycin that permeated and deposited into the skin was quantified via a validated UV-HPLC analytical method. Approximately 34.46 ± 11.317 μg of vancomycin permeated through the skin into the reservoir compartment, whereas approximately 9.88 ± 0.572 μg of vancomycin was deposited in the skin. Approximately 35% of the loaded vancomycin permeated the reservoir compartment, and approximately 10% remained in the skin after 24 h of application.

These findings are in accordance with previously reported data indicating that the amount of permeated vancomycin from MNs to the receiver compartment was more than two times greater than the amount of deposited vancomycin in the skin [42]. This difference might be due to the hydrophilicity of vancomycin and the polymers used in the MN formulation. Sodium alginate and PVA are biodegradable polymers that are intrinsically hydrophilic and may aid in the dissolution, deposition, and permeation of vancomycin across the skin layers [20,43]. The delivery of vancomycin via MNs has been previously investigated by several researchers. For example, vancomycin MNs made of dissolvable polymers (PVA and sodium hyaluronate) showed high drug penetration across porcine skin upon conducting an ex vivo skin permeation study using a Franz cell diffusion apparatus [43]. In a recently published study, sodium alginate was used to fabricate MNs loaded with clindamycin for skin infection [19]. MNs are made by casting, where sodium alginate and gelatin are the main components of the MNs. Clindamycin MNs exhibited a high drug release percentage, and approximately 90% of the drug was released after the MNs were immersed in an aqueous release medium of PBS for 24 h. This release pattern was due to the hydrophilicity and biodegradability of sodium alginate [44].

### 4.4. Evaluating Antimicrobial Activities

#### 4.4.1. Determination of the MIC and MBC Values of Vancomycin

The antimicrobial activity of vancomycin was evaluated in terms of the MIC and MBC against *S. aureus*, *S. epidermidis*, and MRSA, as these are the most common bacteria related to skin infections. According to the data presented in Table 2, vancomycin had inhibitory and biocidal effects on all three tested bacteria. Both MRSA and *S. epidermidis* had twofold greater MICs than did *S. aureus*. However, *S. epidermidis* demonstrated the highest biocidal concentration, with an MBC value equal to 25 µg/mL, while MRSA was the least bactericidal at a concentration of 6.25 µg/mL. The reduced bactericidal activity of vancomycin on *S. aureus*, as shown by higher MBC values, may be attributed to the greater amount of cell wall anchored (CWA) surface proteins relative to the other bacterial species investigated, resulting in an overall decrease in the number of binding sites for vancomycin to bind to the D-Ala-D-Ala C-terminus of the peptidoglycan layer [45,46]. Nonetheless, and according to the National Comprehensive Cancer Study (NCCLS) guidelines, all staphylococcal strains are considered susceptible to vancomycin, as all the tested bacteria exhibited MIC values lower than 4 µg/mL [47].

#### 4.4.2. Evaluating the Antimicrobial Activities of the Vancomycin MNs

To evaluate the antimicrobial activity of the vancomycin MNs, disc diffusion assays were used to monitor the zone of inhibition of vancomycin as proof of the release of vancomycin from the sodium alginate MNs. The test was performed against three staphylococcal species in comparison to free MNs without antibiotics. As shown in Table 3, vancomycin-loaded MNs demonstrated promising antibacterial activity against all three microorganisms, as proven by the ZOI values. The inhibition zone diameters were 6.3 ± 1.5, 7.3 ± 2.3, and 5.0 ± 0 mm for *S. aureus*, *S. epidermidis*, and MRSA, respectively, indicating that the growth of the tested bacteria was successfully inhibited by our vancomycin MNs, as evidenced by the release of vancomycin from the sodium alginate MNs. This finding is consistent with our release studies described earlier. Ziesmer and colleagues developed a vancomycin MN array for the local treatment of MRSA SSTIs and compared the results to those of positive vancomycin controls prepared as vancomycin-infused filter papers with dimensions identical to those of vancomycin MNs. Their disc diffusion studies revealed correlated results between the test results and positive controls, with increased diameters and increased vancomycin amounts. The ZOIs against MRSA shown in this set of experiments indicate that antimicrobial activity is attained after the drug has been released upon dissolution and diffusion from the sodium alginate matrix. In contrast, the drug-free MNs did not show any ZOIs, indicating that no antimicrobial activity was attained by the sodium alginate polymer alone. Notably, in the present study, the antimicrobial properties of the MNs were modulated by the payload within the MNs. In contrast, some of our previous work explored the antimicrobial properties of dissolving MNs by using novel polymers that exhibit either antibacterial or antifungal properties [48]. Moving forward, future work could entail modifying sodium alginate to include pendant groups on the polymer that also exhibit antimicrobial properties, thus serving as an antibiotic potentiator that could augment the efficacy of the formulation.

## 5. Conclusions

In this work, we developed sodium alginate MNs loaded with vancomycin as an innovative approach for treating skin infections, specifically MRSA. Using a double-casting method, we successfully fabricated MNs that exhibited high integrity and were capable of effectively penetrating an ex vivo skin model, as confirmed by light microscopy and mechanical testing. MNs demonstrated substantial drug delivery ability, with 35% of the loaded vancomycin permeated through full-thickness neonatal porcine skin and 10% remaining within the skin after 24 h. This results in an overall delivery efficiency of 45%, indicating the potential of MNs to provide effective dosing for antimicrobial therapy. Furthermore, antibacterial activity tests confirmed the potent effects of the vancomycin-loaded MNs against *C. acnes* and *S. aureus*, confirming their suitability as a treatment modality for skin infections. Although the loading of vancomycin into MNs has been reported previously [44], notably, in this work, the antimicrobial potency of vancomycin-loaded MNs against *C. acnes*, *S. aureus*, and MRSA, not only MRSA, was evaluated. Additionally, the MNs presented in this work presented a higher delivery percentage (35% of the loaded vancomycin) than the previously reported dissolving vancomycin-loaded MNs (8% of the loaded vancomycin) [43]. Our findings highlight the promising role of vancomycin-loaded sodium alginate MNs in offering a non-invasive, efficient alternative to conventional delivery methods for managing MRSA-related skin infections.

## Figures and Tables

**Table 1:** Characteristic functional groups of sodium alginate, polyvinyl alcohol, and vancomycin.

Compound	Functional Group	Wavenumber (cm^−1^)
Sodium alginate	COO	1591
	C-OH	1401
	C-O	1035
Polyvinyl alcohol	C=O	1699
	C-O-C	1246
	C-O	1035
vancomycin	C=O	1651
	C=C	1488
	phenols	1225
	C-O	1035

**Table 2:** MIC and MBC values of vancomycin against *S. aureus, S. epidermidis,* and MRSA.

Bacteria	MIC (µg/mL)	MBC (µg/mL)
*S. aureus*	0.78	12.5
*S. epidermidis*	1.56	25
MRSA	1.56	6.25

**Table 3:** The zone of inhibition (mm) of vancomycin MNs against the tested bacteria correlated with that of the unloaded MNs.

Bacteria	Zone of Inhibition (mm)
	Vancomycin MNs
*S. aureus*	6.3 ± 1.5
*S. epidermidis*	7.3 ± 2.3
MRSA	5.0 ± 0

Free MNs showed no ZOI against any of the tested microorganisms.

## Data Availability

The original contributions presented in the study are included in the article. Further inquiries can be directed to the corresponding authors.
